# An Inline System for Air-in-Line Detection and Automatic Occlusion in Peripheral Intravenous Therapy

**DOI:** 10.7759/cureus.103291

**Published:** 2026-02-09

**Authors:** Drin Rrmoku

**Affiliations:** 1 Electrical Engineering, Independent Research/Practice, Prishtina, ALB

**Keywords:** air-in-line detection, automatic occlusion, healthcare technology, infusion therapy, inline medical device, intravenous safety, patient safety

## Abstract

The presence of air within intravenous (IV) infusion tubing represents a recognized safety concern in routine peripheral IV therapy, particularly when infusion sources run dry or lines are left unattended. While air-in-line detection systems are available in clinical practice, these approaches commonly involve alarm-based notification following detection events. This alarm-dependent approach leaves a gap in automatic mitigation and end-of-infusion control during common clinical workflows.

This work presents the design and operating principle of a compact, low-cost, battery-powered air-in-line detection system intended for peripheral IV tubing. The device employs an infrared-based sensing approach to continuously monitor tubing for the presence of air and for conditions consistent with end of infusion, such as depletion of the fluid source. Upon detection, the system automatically actuates a mechanical occlusion mechanism to halt flow while simultaneously providing notification to clinical staff.

Benchtop evaluation of the proposed system demonstrated a consistent change in infrared sensor output when air was present within standard intravenous tubing compared to fluid-filled conditions. Detection of air-in-line or end-of-infusion events triggered the control logic, resulting in activation of a user notification and engagement of the motor-driven clamping mechanism designed to interrupt flow. These observations support the feasibility of integrating optical air-in-line detection with automatic flow occlusion within a compact, pump-independent inline system.

Existing air-in-line detection approaches typically involve alarm generation to notify clinical staff. The proposed system addresses this limitation by combining continuous monitoring with automatic physical interruption of flow, providing an additional layer of safety and an automated end-of-infusion shutoff mechanism. This approach has the potential to improve IV safety and reduce clinical workload during routine peripheral intravenous therapy.

## Introduction

Air entry into intravenous (IV) infusion tubing is a recognized safety concern in clinical practice and can occur during both invasive procedures and routine peripheral IV therapy. While small amounts of venous air are often tolerated, unintended air-in-line events are undesirable and may pose risk depending on patient condition, infusion context, and response time. Such events may arise from improper priming, disconnected access ports, or infusion sources running dry, allowing air to be drawn into the tubing and progress toward the patient if not promptly addressed [1-3].

In non-intensive care settings such as emergency departments and general wards, intravenous fluid therapy is frequently administered under conditions of high workload and intermittent supervision. Multiple studies and national guidelines have documented variability and suboptimal practices in IV fluid monitoring outside of critical care environments, including delayed recognition of empty infusion sources and inconsistent documentation of infusion status [4-6]. These workflow limitations increase reliance on manual oversight and heighten the likelihood that air-in-line conditions or end-of-infusion events may go unnoticed for extended periods.

Current technological approaches to mitigating air-in-line risk primarily rely on infusion pumps equipped with integrated sensors that generate audible or visual alarms once air has already entered the tubing. While effective in many scenarios, these systems are typically costly, limited to pump-based workflows, and fundamentally dependent on clinician intervention to resolve alarms [7,8]. Alarm burden associated with infusion devices has also been shown to contribute to alarm fatigue, workflow disruption, and delayed responses, particularly in busy clinical environments [9,10]. As a result, existing solutions emphasize detection and notification rather than automatic mitigation.

There remains a practical gap for a supplemental, inline safety mechanism that can operate independently of infusion pumps, continuously monitor IV tubing during routine peripheral infusions, and actively interrupt flow when air-in-line or end-of-infusion conditions are detected. Addressing this gap requires an approach that prioritizes simplicity, portability, low cost, and compatibility with existing IV setups, while reducing reliance on immediate staff intervention.

The objective of this study is to describe the design and operating principle of a compact, low-cost air-in-line detection device intended for routine peripheral intravenous therapy. The proposed system continuously monitors IV tubing for the presence of air and for conditions consistent with depletion of the fluid source. Upon detection, the device provides notification to clinical staff while simultaneously actuating a mechanical occlusion mechanism to halt fluid flow. By combining real-time monitoring with automatic physical interruption of the infusion line, the system is designed to enhance IV safety and provide an automated end-of-infusion shutoff mechanism that supports clinical workflow.

## Technical report

The proposed device is a compact, inline module designed to attach externally to standard peripheral IV tubing. Its primary functions are continuous air-in-line monitoring and automatic interruption of fluid flow upon detection of air or end-of-infusion conditions. The system operates independently of infusion pumps and does not require modification of existing IV equipment.

The air-in-line detection approach is based on changes in optical transmission through standard intravenous tubing. In the proposed configuration, the IV tube is positioned between an infrared light emitter and a corresponding phototransistor receiver. Under normal infusion conditions, the tubing is filled with liquid, and the optical path between the emitter and receiver is characterized by relatively consistent transmission due to the refractive and scattering properties of the fluid. When air enters the tubing, the optical characteristics of the medium within the tube change, resulting in altered light transmission and increased scattering at the fluid-air interfaces. This produces a measurable change in the amount of infrared light received by the phototransistor. The system continuously monitors these variations in received signal level to distinguish between liquid-filled and air-containing segments of the tubing.

**Figure 1:**
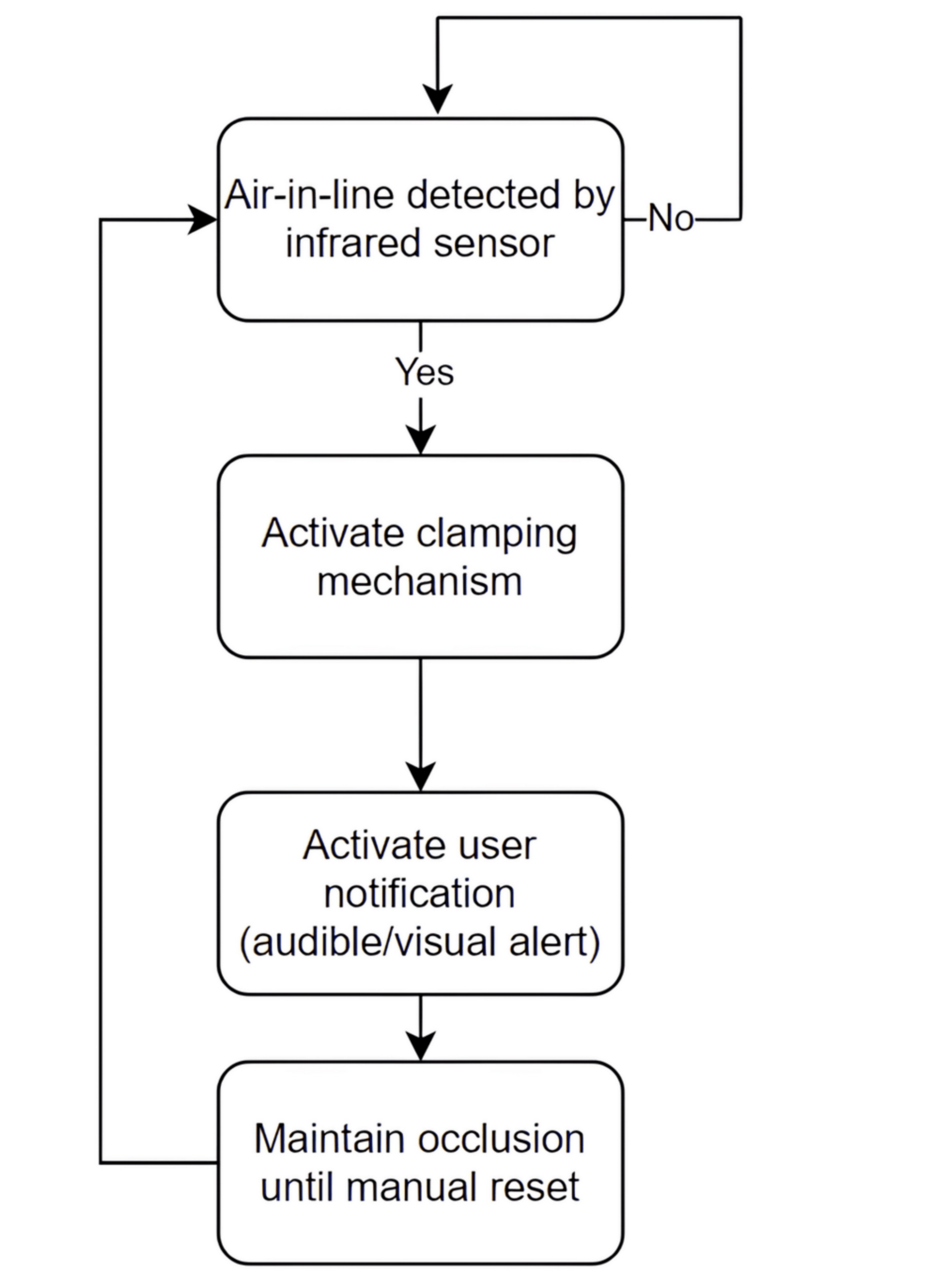
Control logic of the air-in-line detection and automatic occlusion system

Figure 1 illustrates the control logic governing air-in-line detection, automatic flow interruption, user notification, and manual system reset.

**Figure 2:**
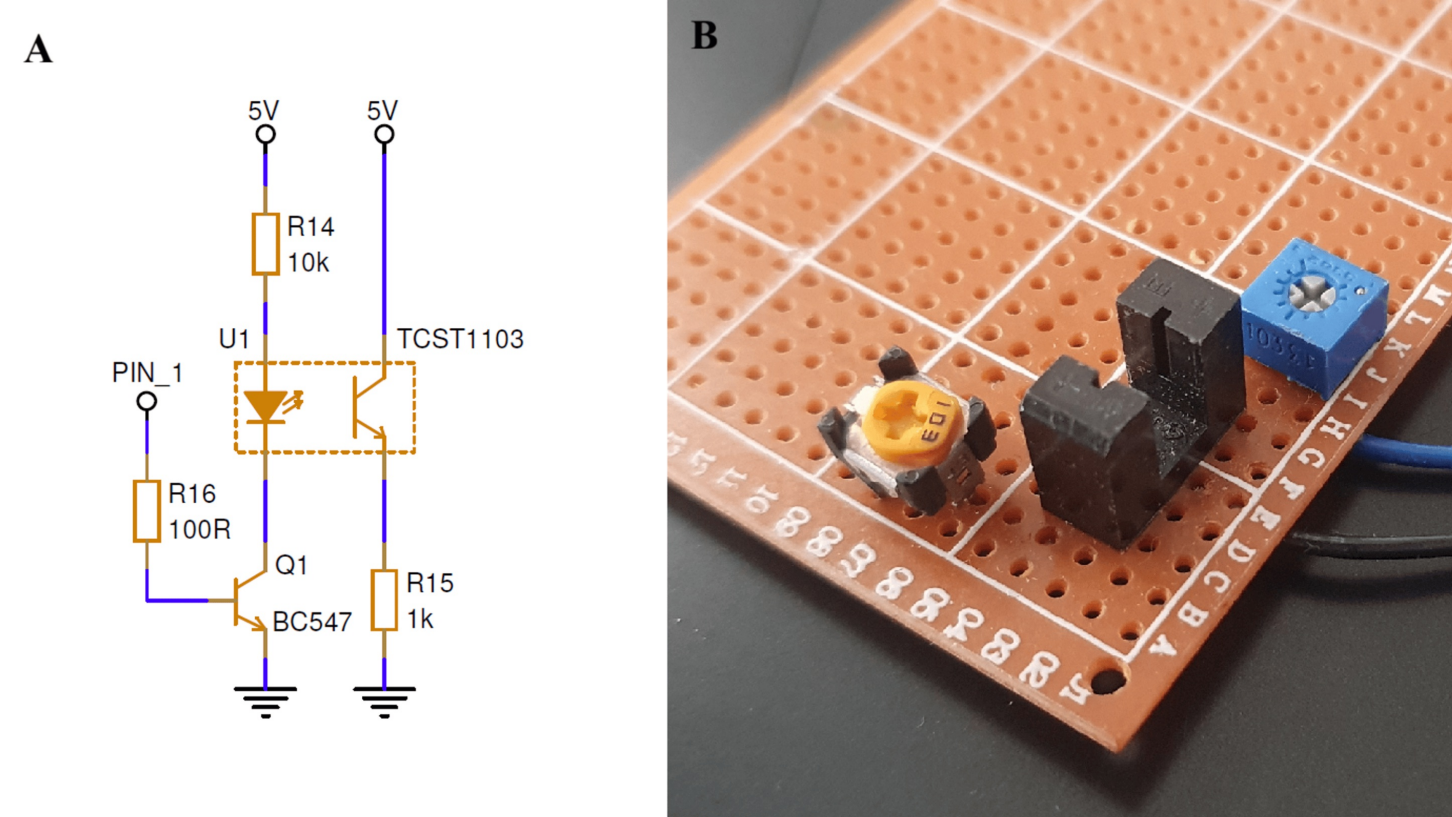
Infrared sensing subsystem schematic and prototype implementation (A) Electrical schematic of the infrared sensing subsystem used for air-in-line detection, illustrating the phototransistor-based signal conditioning and interface to the control unit. (B) Prototype implementation of the infrared sensor components assembled on a perforated board for benchtop evaluation and system integration.

Figure 2 presents the infrared sensing subsystem, including the electrical schematic and a prototype implementation. The infrared emitter is driven through transistor Q1, which allows the infrared emitter to be periodically enabled and disabled under control of the system logic, avoiding continuous illumination and reducing overall power consumption.

**Figure 3:**
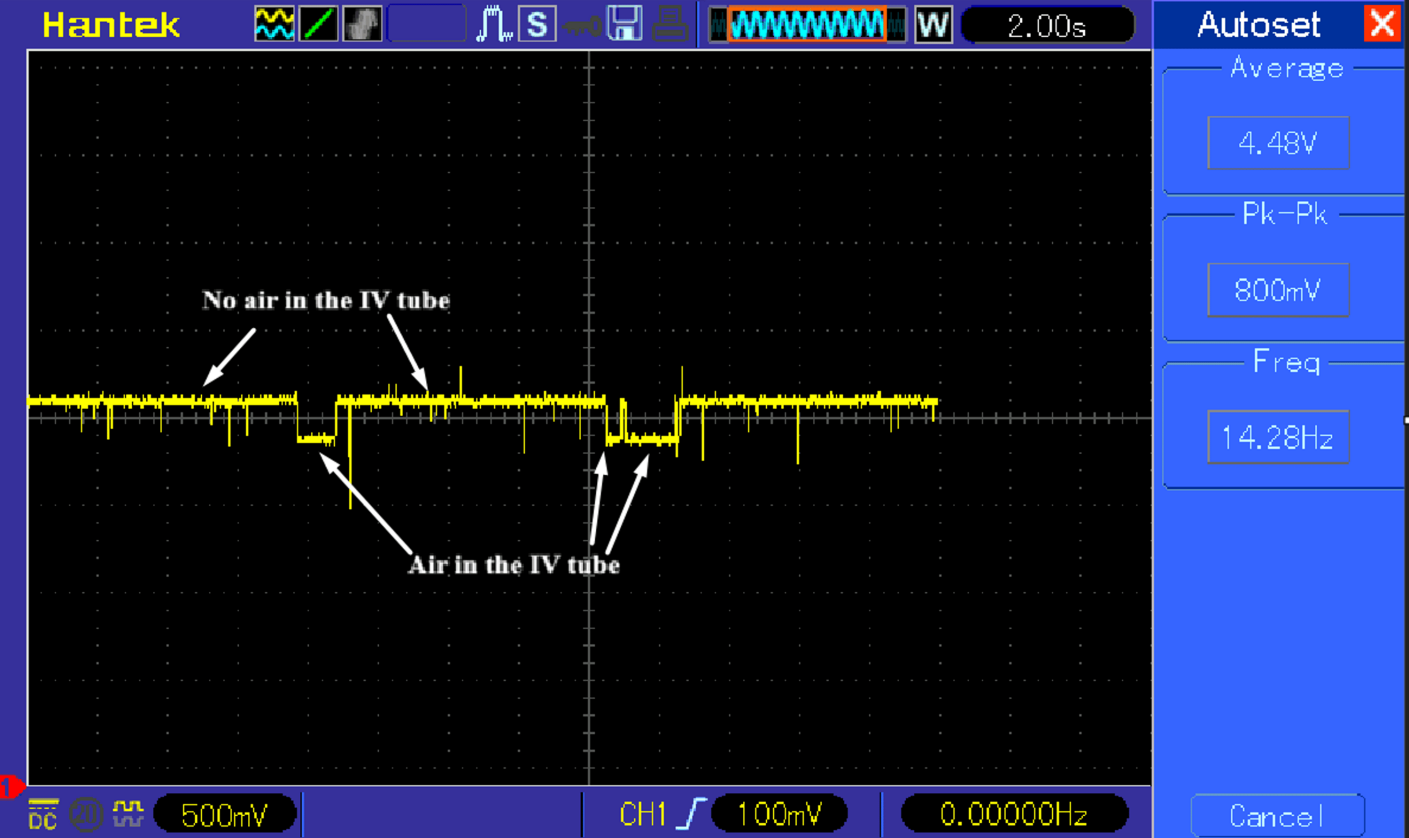
Oscilloscope measurement of infrared sensor output during air-in-line detection

Figure 3 presents an oscilloscope measurement of the infrared sensor output signal during benchtop evaluation. A distinct change in signal level is observed when air is present within the intravenous tubing compared to normal fluid-filled conditions.

**Figure 4:**
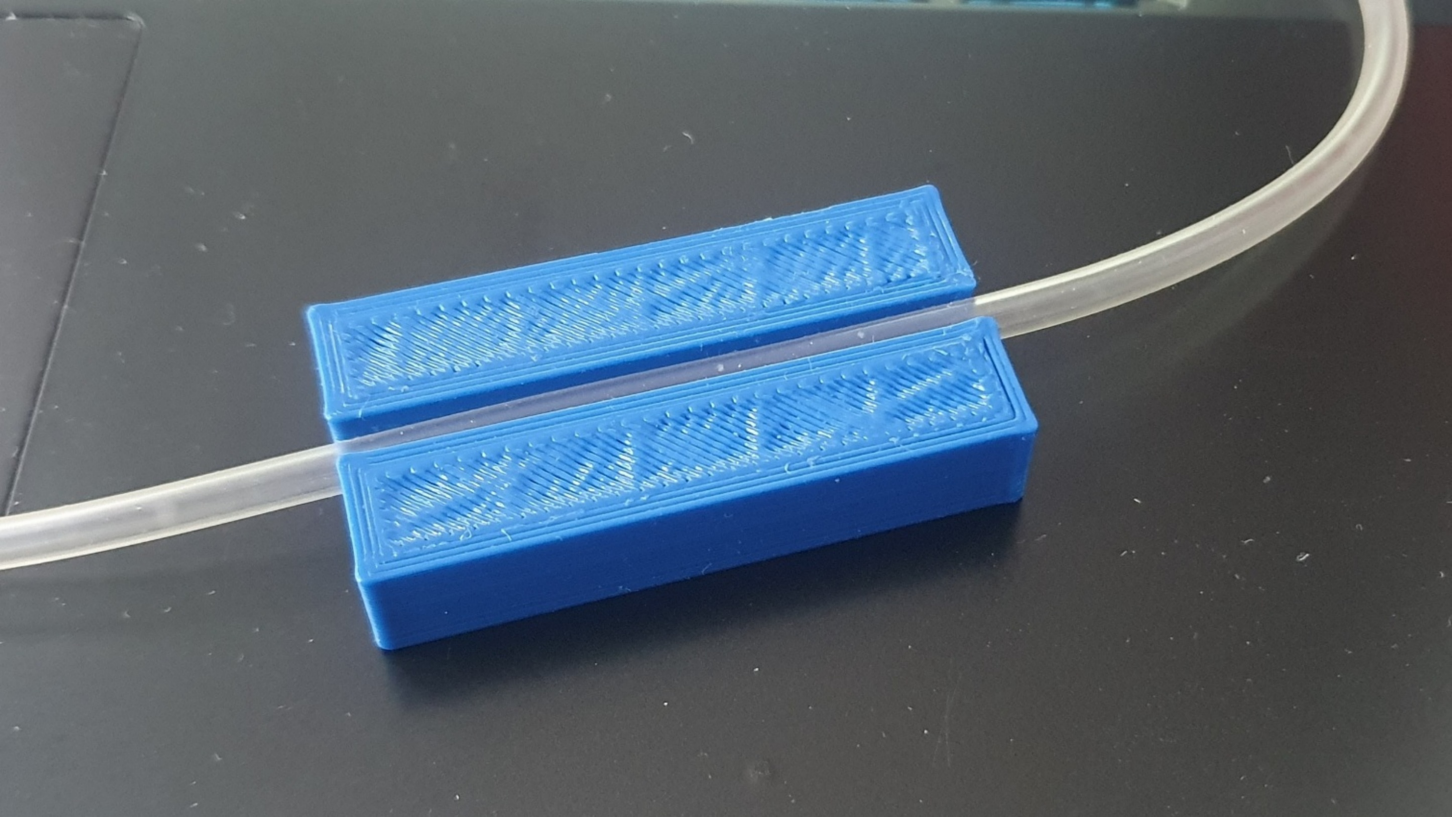
Press-fit mechanical integration concept for inline IV tubing sensing

Figure 4 presents the prototype of a press-fit mechanical housing used to position standard intravenous tubing between the infrared emitter and receiver. The tubing is inserted directly into the device without cutting or modifying the IV line, enabling upgradable integration with existing infusion setups. The two-piece housing maintains consistent tube alignment for optical sensing while allowing straightforward insertion and removal during setup.

Upon detection of air-in-line or end-of-infusion conditions by the infrared sensing subsystem, a low-power microcontroller initiates two actions in parallel: activation of a user notification and actuation of a mechanical clamping mechanism. An audible alert is generated to inform clinical staff that an air-related event has been detected, while the clamping mechanism is simultaneously engaged to physically interrupt flow within the intravenous tubing. Flow interruption is achieved using a compact N20 DC motor coupled to a geared transmission, which provides the increased torque required to reliably compress standard IV tubing. The motor-driven clamp applies sufficient force to occlude the tubing and halt both liquid and air flow. Once activated, the clamping mechanism remains engaged until manually reset by the user, ensuring that flow cannot resume unintentionally following an air-in-line or end-of-infusion event.

**Figure 5:**
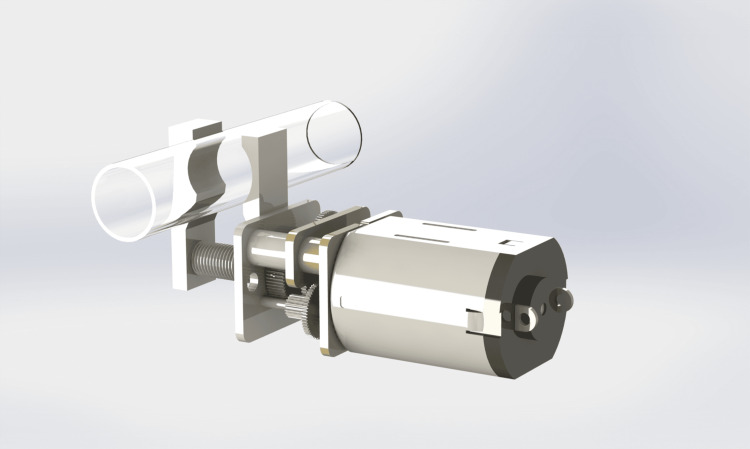
Motor-driven clamping mechanism for automatic interruption of IV flow Image: CAD rendering created by the Author

Figure 5 illustrates the proposed clamping mechanism, in which a compact geared DC motor drives a mechanical assembly that compresses the intravenous tubing to interrupt flow.

**Figure 6:**
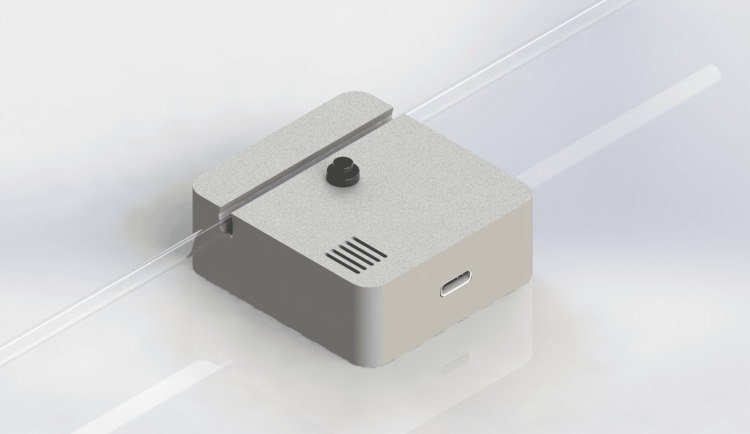
Rendering of the integrated inline air-in-line detection and occlusion device Image: CAD rendering created by the Author

Figure 6 presents a conceptual rendering of the integrated inline device, illustrating the overall form factor and intended placement on standard intravenous tubing. The device is designed to attach externally to existing IV tubing without requiring cutting, splicing, or modification of the infusion line, enabling upgradable deployment as an add-on safety mechanism. This integrated design emphasizes portability, simplicity of use, and automated flow interruption upon detection of air-in-line or end-of-infusion conditions.

## Discussion

The findings of this work demonstrate that changes in optical transmission through standard peripheral intravenous tubing can be used to reliably identify air-in-line and end-of-infusion conditions under benchtop evaluation. The observed and repeatable difference in infrared sensor output between fluid-filled and air-containing tubing segments indicates that continuous external monitoring is feasible without direct integration into infusion pumps. The consistent triggering of both user notification and flow occlusion following detection supports the functional viability of combining air sensing with automatic mitigation in a compact inline format.

In routine clinical practice, air-in-line detection is most commonly addressed through pump-integrated alarm systems that alert clinicians after air has entered the infusion line [4,8-10]. While effective in many settings, these systems depend on timely staff response and are susceptible to alarm fatigue, particularly in non-intensive care environments where IV therapy is frequently administered under intermittent supervision [5,9,10]. The approach presented in this study addresses this limitation by introducing an automatic physical interruption of flow immediately following detection, reducing reliance on immediate clinician intervention and potentially limiting downstream air propagation during unattended infusions or when fluid sources become depleted.

The pump-independent and externally attachable nature of the proposed system is clinically relevant for peripheral IV workflows where advanced infusion pumps or integrated air-detection features may not be available. By operating as an add-on safety mechanism that does not require modification of existing tubing or infusion equipment, the system is conceptually suited for broader deployment across diverse care settings, including emergency departments, general wards, and resource-limited environments [4,8]. This positioning distinguishes the proposed approach from existing alarm-only strategies by emphasizing automated response and compatibility rather than system integration complexity [8-10].

Several limitations should be acknowledged. The current evaluation was limited to benchtop testing and did not involve human subjects or clinically representative infusion scenarios. Performance under varying flow rates, tubing materials, ambient lighting conditions, and prolonged clinical use was not assessed. As such, while the results support feasibility, further validation under controlled and real-world conditions is required to establish robustness, reliability, and clinical impact. Future work should focus on systematic validation and refinement of the device architecture to support safe translation into clinical environments.

## Conclusions

This work presents the design of a compact, pump-independent inline system for air-in-line detection and automatic flow interruption in peripheral intravenous therapy. The proposed approach integrates optical sensing, low-power control logic, and user notification within an externally attachable form factor, addressing a gap between passive air detection and active safety response in standard infusion setups. Unlike alarm-only solutions, the system is intended to provide an immediate automated response following detection while remaining independent of infusion pumps or proprietary hardware.

The device is designed for use with existing intravenous tubing without modification, supporting straightforward deployment as an add-on safety measure across a range of clinical environments. By emphasizing compatibility, compactness, and low-power operation, the system aims to support broader adoption in settings where advanced infusion pumps or integrated air-detection features may not be available. Future work will focus on controlled validation of system operation under clinically representative infusion conditions and further refinement of the overall device architecture.
